# Implementing Introductory Training in Trauma-Informed Care Into Mental Health Rehabilitation Services: A Mixed Methods Evaluation

**DOI:** 10.3389/fpsyt.2021.810814

**Published:** 2022-01-20

**Authors:** Laura Nation, Nicola Spence, Stephen Parker, Maddison Paige Wheeler, Kate Powe, Mei Siew, Tamara Nevin, Michelle McKay, Michelle White, Frances Louise Dark

**Affiliations:** ^1^Metro South Addiction and Mental Health Services, Brisbane, QLD, Australia; ^2^Faculty of Medicine, The University of Queensland, Herston, QLD, Australia

**Keywords:** trauma-informed care, training, recovery orientated mental health rehabilitation, implementation, competency framework

## Abstract

**Objective:**

This paper describes the implementation of training in trauma-informed care (TIC) across a mental health rehabilitation service.

**Method:**

A mixed-methods approach was applied incorporating baseline measures of staff attitudes toward TIC, quantitative description of staff training participation, and semi-structured interviews of Team Leaders' views on the implementation of TIC.

**Results:**

Fifty-five of 123 staff responded to the Organizational Change Readiness Assessment (OCRA) survey (44.7%). Training completion varied considerably between the eight rehabilitation teams (4.8–78%). Analysis of the Team Leader interviews identified four broad themes: The need to respect the person's life journey including the risk of re-traumatization; the importance of considering the context of implementing TIC training; TIC being an essential part of mental health care; and staff may also have trauma histories.

**Conclusions:**

Staff working in mental health rehabilitation are supportive of the need for TIC. The variable training uptake did not reflect the staff comments about the importance of TIC. The burden of adjusting mental health care delivery to COVID-19 restrictions was reported as a major influence on the uptake of training. Systematically implementing training in TIC is required but needs to be complemented by a structured organizational approach to aid embedding this approach into daily mental healthcare delivery.

## Introduction

A history of experiencing trauma is common in people seeking help from mental health services (1, 2). The pervasiveness of trauma and its impact on the development; presentation; and management of people experiencing mental illness is well established. The Australian National Framework for Recovery-Oriented Mental Health Services (3) was endorsed in 2013 and emphasizes the importance of Trauma-Informed Care (TIC).

There are multiple definitions of trauma. The Substance Abuse and Mental Health Services Administration (SAMHSA) reviewed the definitions and developed the following concept:

“Individual trauma results from an event, series of events, or set of circumstances that is experienced by an individual as physically or emotionally harmful or life threatening and that has lasting adverse effects on the individual's functioning and mental, physical, social, emotional, or spiritual well-being” (4). Trauma can follow acute single events or result from accumulative traumas over a lifetime. Interpersonal trauma is particularly common in people seeking or requiring mental health care. Sometimes trauma is not the presenting mental health complaint, and it can often be missed. The secondary manifestations of trauma may be the primary focus of care (e.g., substance misuse, interpersonal difficulties, paranoia) and the link to a trauma history can go unrecognized. Principles of TIC have been developed emphasizing the importance of safety, trustworthiness, choice, collaboration and empowerment (4). These principles can provide a universal practice framework for mental healthcare professionals and help to ensure services/professionals are sensitive to the heterogenous manifestations of trauma and adapt their practice to reduce the risk of re-traumatisation (5).

TIC involves a systems approach and staff awareness rather than a sole focus on providing a specific group or individual trauma therapy (6). Transitioning toward TIC requires organizational change within mental health services and staff training and support (5, 7). The literature recommends a multi-level (trauma informed, trauma skills, trauma enhanced, and trauma specialist) competency-based training structure (4). The fundamental competency level is being trauma informed which is relevant to all staff (4, 8, 9).

Training alone is unlikely to lead to the change in practice required to embed TIC into standard care (10, 11). There is also a need for an organizational response that has TIC as part of routine clinical practice and organizational governance systems (12). Recent commentaries have emphasized the challenges of implementing TIC, especially in public mental health services (7, 12). Barriers to implementing TIC within mental health services include resourcing, organizational culture, the dominance of the bio-medical model, and clinician misconceptions (13). Isobel notes there can be a lack of staff clarity of what is meant by trauma and issues in sustaining attempts at implementing TIC (7). Despite the recognized need there is limited evidence to guide the effective implementation of Trauma Inform Care (11).

Specialized mental health rehabilitation services aim to provide environments that facilitates recovery. However, the manifestations of trauma can influence therapeutic engagement and become a limiting factor in optimal personal recovery (2, 14). This study evaluates a quality improvement activity of implementing introductory, online trauma-informed training across the eight rehabilitation teams of an Australian mental health service. The training aims to increase knowledge and awareness of trauma and the impact of trauma on individuals.

## Objectives

The primary objective was to evaluate the organizational goal to have all staff working in the rehabilitation teams trained in TIC. In addition, Team Leaders views on the integration of TIC into routine clinical care was explored.

## Methods

### Study Design

A mixed-methods approach was applied, this incorporated baseline measures of staff attitudes toward TIC, quantitative description of staff training participation, and semi-structured interviews of Team Leaders' views on the implementation of TIC.

The study and its findings were reported considering the COREQ checklist (15) (see Supplementary Material). All participation was based on voluntary informed consent, and ethics approval was obtained before study commencement (Metro South Human Ethics Committee ethical clearance HREC/2019/QMS/52067).

### Study Population

The study population included clinicians (occupational therapists, psychologists, social workers, psychiatrists, psychiatrists in training, nursing staff), rehabilitation therapy aids, and peer workers of the eight rehabilitation teams of a large metropolitan public mental health service (*n* = 123) (Table 1) and Team Leaders (*n* = 8) at these services who are responsible for operational management of the teams and operational supervision of staff.

**Table 1:** Rehabilitation team descriptions and training completion rates.

**Rehabilitation service component**	**Team site**	**Staff** **(*N*)**	**Training completion**	
			***n***	**%**
Early Psychosis team^a^	PAH	9	1	11%
Extended treatment and rehabilitation^b^	Bayside	17	9	53%
Community Care Unit^c^	Bayside	24	6	25%
	Coorparoo	16	1	6.3%
	Logan	21	1	4.8%
Mobile Intensive Rehabilitation Team^d^	Logan	13	1	6.3%
	PAH	14	2	14.3%
Transitional Housing Team^e^	PAH	9	7	78%

a *Intensive community-based support to 18–25-year-olds within 2 years of the first onset of psychotic symptoms (16)*.

b *Sub-acute medium-term mental health inpatient mental health rehabilitation for adults aged 18–65 years (17)*.

c *Community-based recovery-oriented residential mental health rehabilitation support focusing on living skills development and community integration (18)*.

d *Community-based intensive mental health rehabilitation support based on an adaptation of the Assertive Community Treatment. model (23)*.

e *Provides transitional accommodation in community-based independent living units, and intensive domestic and community support to consumers for up to 6 months*.

### Study Setting

The study was undertaken in a large metropolitan public mental health service in Queensland (Australia) whose catchment area includes over 1.1 million people. An extensive range of mental health services are provided, including eight rehabilitation teams. The rehabilitation teams include a team supporting people with a history of homelessness (Transitional Housing Team), two community-based outreach teams (Mobile Intensive Rehabilitation Teams), an Early Psychosis team and three residential rehabilitation units (Community Care Units) (see Table 1). The senior leadership across the rehabilitation service identified the implementation of TIC as a quality improvement activity. The implementation experience reported in this paper covered the period 15/11/2019 to 2/4/2021. This period was inclusive of unanticipated disruptions in service activity due to COVID-19 commencing in March 2020.

### TIC Online Training

**Figure 1:**
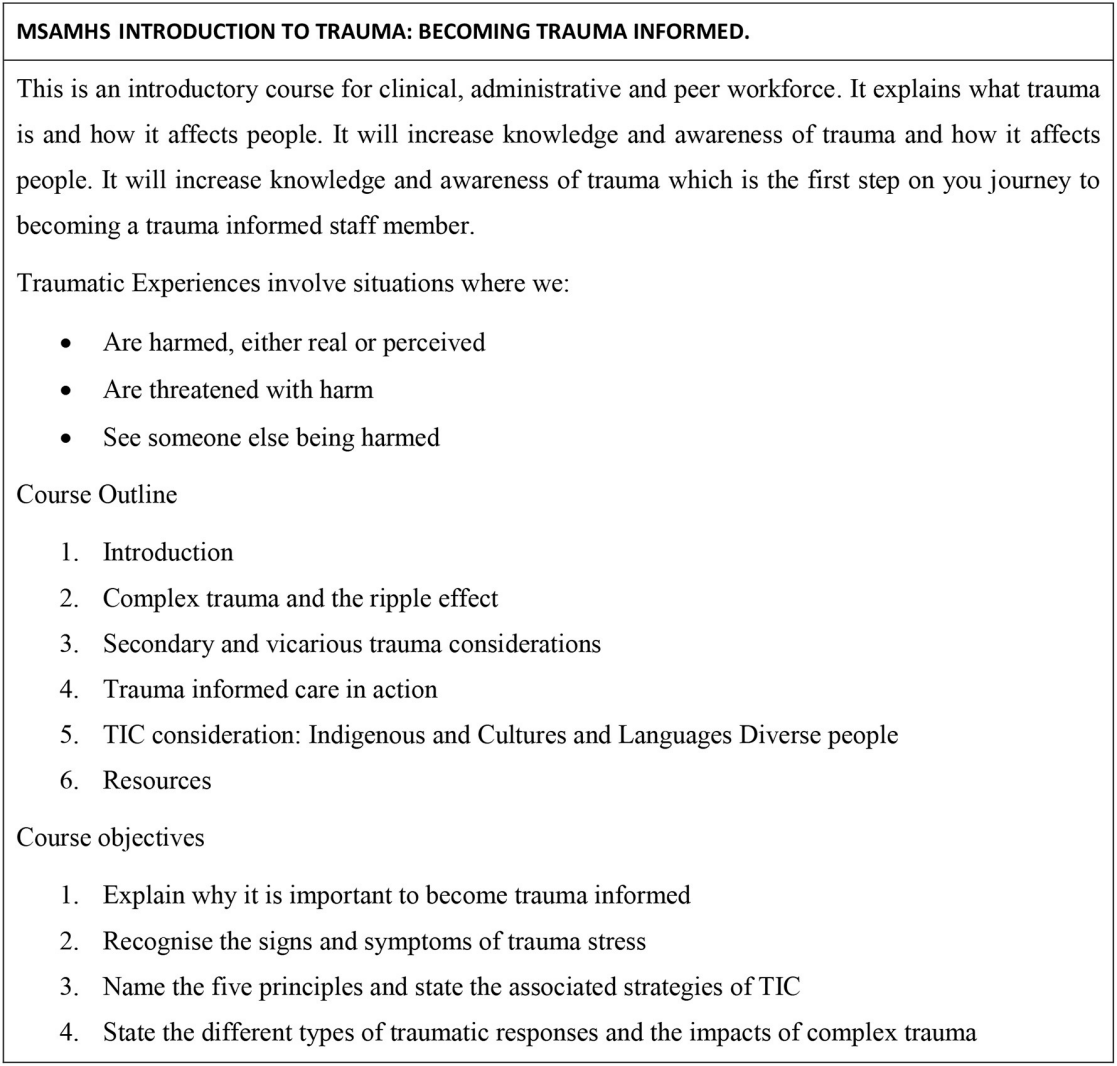
MSAMHS introduction to trauma: becoming trauma informed. Metro south addiction and mental health services (2020). MSHLearn (birchlp.com.au).

The service's multi-professional Psychological Trauma Steering Committee developed the introductory online staff training (19). Figure 1 outlines the focus and content of the online training.

### Implementation Plan

**Figure 2:**
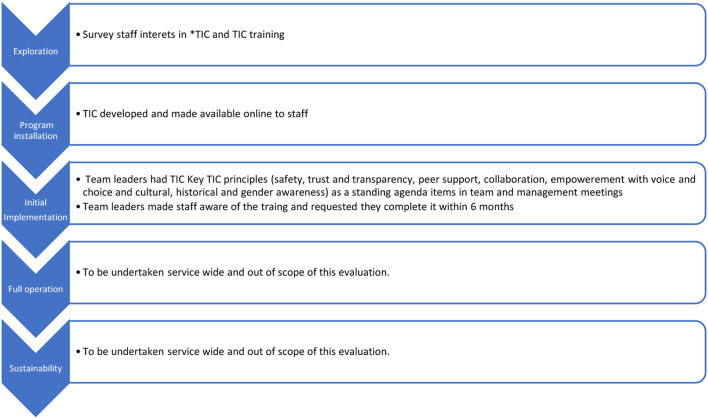
Five stages of implementation (20) (The first 3 stages were evaluated in this study). TIC, Trauma informed care.

The implementation plan focused on the first three stages of the five stages of implementation as described by Fixsen et al. (13) (Figure 2). In the exploration phase, staff were surveyed to ascertain their perception of the organizational readiness to introduce TIC training. The survey results influenced the initial implementation phase, which focused on staff training and incorporating the TIC principles into multidisciplinary team meetings and staff supervision. Team Leaders audited training completion within their teams. Key TIC principles (Figure 2) were made a standing item within professional and operational supervision in the rehabilitation teams to facilitate applying the training to practice. After 6 months, Team Leaders were interviewed via phone or teleconference to ascertain their views on implementing the TIC training into clinical practice. The implementation stages 4 (full implementation), and stages 5 (sustainability) were out of scope of this study.

## Measures

### Organizational Change Readiness Assessment

There are many organizational change measures available often with limited evaluation of their psychometric properties (20). The reliability and validity of instruments is context dependent. In this study we aimed to find an instrument that: (1) could be adapted for the public rehabilitation service context, (2) that was short to encourage completion, and (3) that focused on staff perceptions of the planned implementation of TIC. The instrument was developed in Canada (21) and designed to provide a quick overview of factors potentially inhibiting or facilitating change that may need to be addressed in a change process.

The OCRA (21) profiles an organization's “change readiness” from the perspective of staff using 29 questions. It can be adapted to focus on a particular initiative. The instrument provides a profile of service direction, operations and support system levers that facilitate change or may hinder change (Table 2). Each lever has a number of subcategories rated from 1 (most supportive) to 5 (most inhibitory). Ratings 1–3 indicate there is a likelihood that these levers and subcategories are likely to support change and rating of 4 or 5 are likely to inhibit change (Table 2). No psychometric data is available on this instrument.

**Table 2:** Organizational change readiness assessment profile.

		**Scoring** ^**a**^				
		**1**	**2**	**3**	**4**	**5**
**Lever**	**Questions**	**Likely to support**			**Likely to hinder**	
Service direction	a. External environment			X		
	b. Leadership			X		
	c. Strategic direction				X	
Operations	d. Organization structure, tasks, work processes				X	
	e. Management processes and communication				X	
Support systems	f. Culture, norms, morale			X		
	g. Human resource systems				X	
	h. Employees' personal goals and competencies				X	
	i. Information processes				X	

a *1 = most supportive of change, 5 = most inhibitory of change*.

### Team Leader Interview Questions

**Figure 3:**
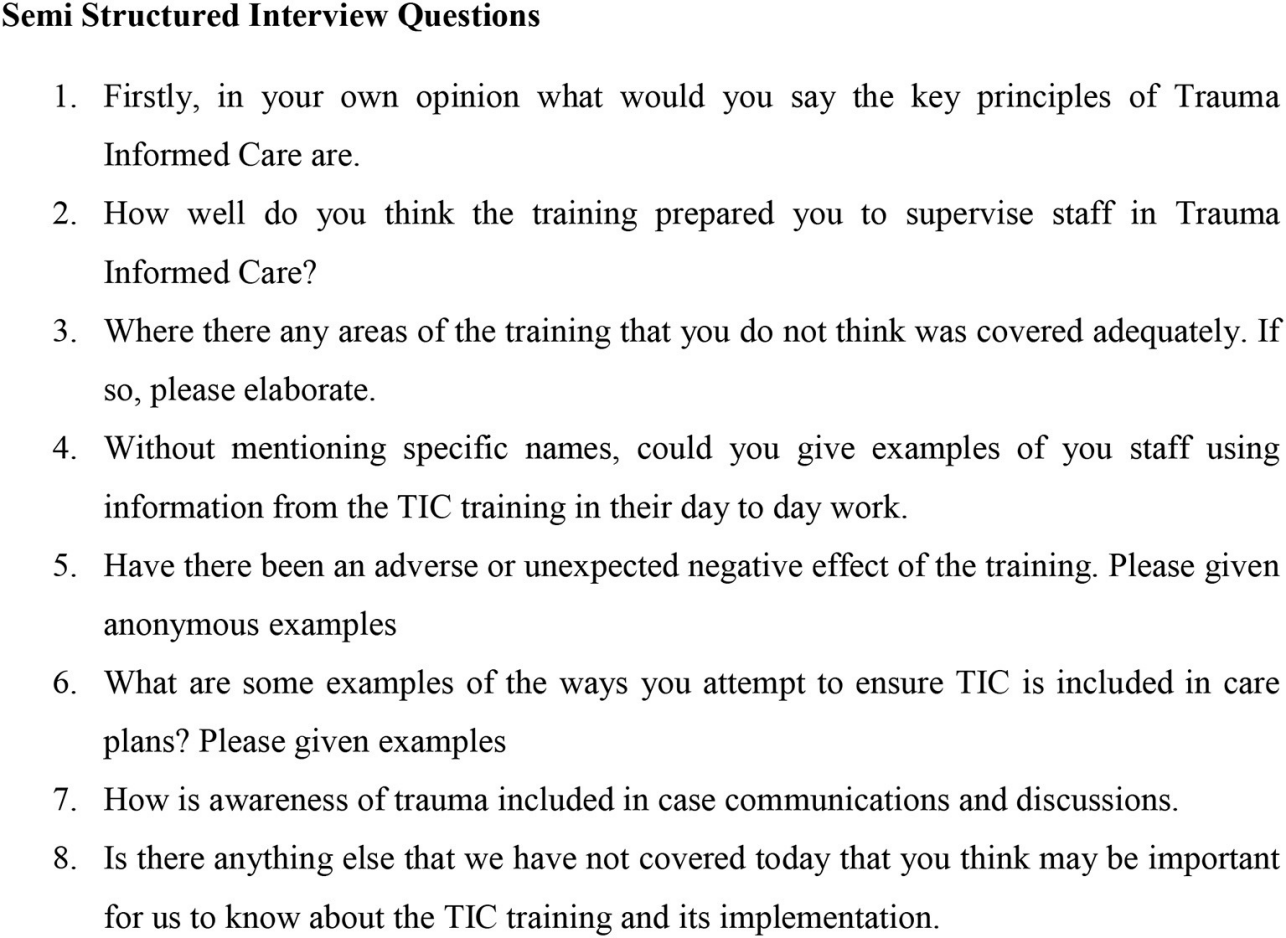
Team Leader interview questions.

The interview comprised eight open-ended questions developed by the authors and designed to capture information about Team leaders' views on the implementation of TIC into practice (Figure 3). Due to COVID restrictions all interviews were conducted either by telephone or videoconferencing with notes taken by the interviewer. Participants were given the option of having the interview conducted by an independent research assistant or the Director of the Rehabilitation services; all chose the Director.

### Analysis

The OCRA scoring scheme (21) was applied in profiling the survey results. The de-identified Team Leader interview transcripts were analyzed from an inductive thematic approach following the steps of Braun and Clarke (22) using the NVIVO12 program. These steps were not linear but provide elements of a recursive analysis of the data. Two authors were the main data coders who familiarized themselves with the data before coding each item and collating the codes and data extracts. The themes were reviewed with reflection on representing and synthesizing the data identified in the analysis. Two authors completed this thematic analysis independently and then worked collaboratively with another author to get consensus on the final themes and illustrative transcript extracts. Participants did not provide feedback on the findings.

## Results

### Organizational Change Readiness Assessment

Fifty-five of the 123 staff (44.7%) completed the survey. The service profile indicated that possible barriers to implementation were in the lever categories of Service Direction (Strategic direction); Operation (organizational structure, management processes and communication) and Support Systems (human resource systems and employees' personal goals and competencies) (Table 2). The initial implementation plan addressed the identified barriers via the TIC training and Team Leaders prioritizing TIC in management processes. There was a relative strength in the lever categories Service Direction (external environment, and leadership) and Support system levers (service culture, norms, and morale).

### Training Completion Rates

Training completion rates varied between the eight rehabilitation teams (6.3–78%, Table 1). Teams with the highest training uptake had one or more of the authors as program champions working in the team.

### Thematic Analysis of Team Leader Interviews

All eight Team Leaders within the rehabilitation service were interviewed. Below are the themes that emerged through the analysis (Table 3 and Figure 3).

**Table 3:** Break down of the thematic analysis of the semi structured interviews of the Team Leaders.

**Theme**	**Examples/meaning units**
Theme 1. The need for to respect the person's life journey and the risk of retraumatisation	• Aware of the long-lasting impact and influence of historical trauma • Reframe person as an individual rather than diagnosis • What happens to you rather than what is wrong • More holistic • Look longitudinally rather than cross sectionally • It is important we don't repeat traumas inadvertently • Experience trauma from mental health care • Even though you wouldn't know it if (you) break trust even in a little way. Easy to take it for granted. Promised tea before meeting got caught up and forgot. Later apologized
Theme 2. The context of implementing TIC training is important	• (due to) COVID…. (TIC training) had slipped my mind.' • I found myself getting frustrated with staff and residents. • Timely given the year we have had. • Reflect on how your stress has impacted on work. can get brusque. • Don't implement TIC training before a pandemic. • The supervisions and TIC follow up got interrupted by COVID. • Change fatigue esp. in general COVID climate • Staff shortages and change capacity to invest in TIC. • Psychologist won't do sessions in residents' unit if trauma issues may come up
Theme 3. (TIC) is an essential part of mental health care	• TIC part of our philosophy of practice • It is why we do what we do • The principles are that we work with all clients from a care that recognizes trauma in multiple ways, individual, not formulaic. • Without the training and background knowledge easy to be judgemental • TIC helps make services cater and avoid re-traumatization… • Integrate with other work e.g. sensory • Latched onto the similarities with sensory work • Focus on sensory work • Increased request for trauma therapy
Theme 4. Staff may also have trauma histories	• Understand staff may have traumas or have experienced trauma in the workplace • Appreciate staff who have experienced trauma. • Help support staff • The training did not cover the issue of clinicians who themselves maybe traumatized

#### Theme 1: The Need for to Respect the Person's Life Journey and the Risk of Re-traumatisation

Patients referred to specialized mental health rehabilitation teams have ongoing functional impairment related to their mental illness that has not responded to general mental health care. Team Leaders emphasized the need to take an individualized, longitudinal, and holistic perspective with each patient. For example, understanding a person's history and trauma background would allow staff to work collaboratively with the individual to tailor treatments.

*In one case with a male with a history of sexual abuse we ceased the depot because of a concern it could be retraumatizing*. (Participant 3)

…… *you wouldn't know it if you break trust even in a little way. Easy to take it for granted* (Participant 6).

*Our work needs to account for traumas they have suffered and impact on how they look and behave. It is important we don't repeat traumas or inadvertently they experience trauma from mental health care (Participant 1)*.

*Awareness of consumer journey. …..TIC is overarching significance. …. e.g. ….. person who left school early got involved in the correctional system. Need to understand why they respond in circumstances (Participant 2)*.

…*Aware of the long-lasting impact and influence of historical trauma (Participant 4)*.

#### Theme 2: The Context of Implementing TIC Training is Important

Soon after commencing the implementation of TIC training, the impact of the health response to COVID 19 took effect. Team Leaders were central to the COVID 19 response within each team. While Team Leaders recognized the importance of TIC, this was difficult to prioritize given the effects of COVID 19.

*Change fatigue especially in the general COVID climate…. Bombarded by emails… Staff shortages and change capacity to invest in TIC*. (Participant 4).

*Don't implement TIC training before a pandemic. The supervision and TIC follow up got interrupted by COVID* (Participant 1).

#### Theme 3: TIC is an Essential Part of Mental Health Care

There was an awareness and acceptance of the high rates of trauma in the life histories of people with mental illness. Team Leaders viewed trauma as common and believed staff needed to be supported to recognize how trauma can manifest and best assist the person.

*(The Early Psychosis) team uses a trauma perspective as part of core business. Especially relevant as team sees refugee and people from low socioeconomic (groups). Need to differentiate trauma from other processes leading to psychosis* (Participant5)

*The principles are that we work with all clients from a care that recognizes trauma in multiple ways, individual, not formulaic*. (Participant 1)

*TIC is part of our philosophy of practice….It is why we do what we do* (Participant 8).

Staff commented that TIC complemented other mental health care delivered within the rehabilitation setting.

*(TIC) is integrated with other work e.g., sensory. Sensory to deal with functional disabilities (of trauma)* (Participant 1).

#### Theme 4: “Staff May Have Traumas…”

Team Leaders commented that completing the TIC training may be triggering for staff. Equally, they viewed working in mental health may inadvertently retrigger staffs' own past traumas.

*Also, try to understand staff may have traumas or have experienced trauma in the workplace (Participant 1)*.

*Appreciate staff who have experienced trauma. …..Help support staff (Participant 8)*.

### Minor Themes

There were comments arising from four team leaders' interviews that despite not being frequently endorsed were considered to represent themes worthy of inclusion. The rehabilitation teams have a significant proportion of non-clinical staff who may have differing needs in relation to TIC training and practice.

*Differing abilities…depending on whether clinical or non-clinical* (Participant 1).

*There was some fear from the peer workers that they would be expected to do trauma therapy* (Participant 2).

The team leaders are usually senior staff who have extensive clinical experience. The TIC training did not expand their knowledge base and there was a recognition of the need for the development of further training.

*The training ignited existing knowledge. I have been working for several years …I will apply my knowledge in professional supervision and do further reading* (Participant 2).

*I was already aware of the importance of trauma. the TIC training didn't make a lot of personal difference…not new for me* (Participant 3).

*As a psychologist nothing new in the training as TIC is part of our training. More in depth training as a psychologist…. (TIC training) is good basic training, sufficient at this level…need for follow-up training especially for clinicians. In general, we are not meeting the need (for specific trauma therapy)* (Participant 4).

## Discussion

The association of trauma and mental illness is well recognized (1, 2). This study evaluated aspects of the implementation of introductory TIC training into the rehabilitation curriculum of teams within a rehabilitation service of a large public mental health service. Despite the recognition of the importance of trauma this study found variable uptake of TIC training and the initial objectives of implementation were not met.

The OCRA survey results were encouraging regarding organizational readiness and validated the initial implementation plan focusing on staff training and supporting practice change using existing team management structures. However, this instrument measures a point in time and cannot predict emergent issues, particularly external events like the global pandemic.

There was wide variability in the uptake of the training. Teams where the authors worked had the highest levels of training completion. This highlights the influence of program champions in successful change implementation (16). The implementation literature also refers to the influence of timing on successful implementation and the concept of absorptive capacity on how much change an organization can accommodate (16). The Team Leader interviews referred to this issue as they noted the role of staff fatigue due to the demands of the response to COVID-19. Making TIC training mandatory would be an organizational solution that would be congruent with the importance of trauma in mental health.

The minor themes were consistent with the limitations of entry level training that focusses on the fundamentals of TIC but does not meet the needs of a diverse workforce. In addition, there is an identified gap in training staff in other levels of competency e.g., trauma skilled, trauma enhanced and trauma specialist. The current limited ability for this public mental health service to meet the need for specific trauma therapy was seen as a gap in care provision. It is necessary to have a whole of service structured competency framework to address this need. This study investigated the basic level of trauma competency within a section of a large public mental health service. The findings in this study add support for further organizational support and investment to build broad based, multilevel competency levels in staff that is congruent with their roles. This would involve fundamental TIC training for all staff, as well as training to develop staff specialists in specific trauma therapy. A comprehensive organizational response would also include provision for staff supervision and for ongoing training.

Leadership is core to any successful change implementation (16). Team Leaders are critical in facilitating behavioral change in the staff they manage and supervise. The key themes emerging from the qualitative analysis of the Team Leader interviews foregrounded how awareness of trauma is integral to mental health care. Having a trauma lens can influence clinical decisions with the recognition that vigilance is needed to minimize re-traumatization. Taking a person-centered approach was seen as a way to ensure that care especially in relation to trauma is individualized and the uniqueness of the person's experience is appreciated (17).

## Limitations

The authors recognize the need for larger, more methodologically rigorous studies of the effectiveness of strategies for the implementation of TIC. This study examined a service improvement initiative of the rehabilitation teams of a large public mental health service, in the absence of a service wide comprehensive implementation plan. Despite this qualification this study is an example of how services within large organizations can begin to take responsibility for quality activities.

The authors also acknowledge the limitation of the measures (OCRA and the Team Leader questions) not being validated but context specific. The survey response (44.7%) did not represent the views of all staff or staff in all teams. To minimize the potential influence of participant researchers, discipline-specific data on who completed the training was not collected. This meant the authors were unable to comment about training differences between disciplines. Optimal data saturation (18) was not reached for the thematic analysis due to the fixed population of Team Leaders (*n* = 8). Another limitation to this study was the derailment that occurred following COVID-19 with regards both to the response rate from the survey as well as the actual implementation of the training. In this research staff opinions were collected rather than exploring factors other than COVID-19 that impacted on training uptake.

This evaluation does not involve the complete implementation of TIC training which would require endorsement at a senior executive level of the service but focused on the initial stages and will inform ongoing TIC implementation in the service.

## Conclusions

Introductory training in a trauma-informed approach was considered necessary but not sufficient to embed TIC into routine care. Staff also emphasized that an overall organizational commitment is required for meaningful implementation of TIC (9). The need to have a systematic approach that incorporates all strands of TIC and uses a structured competency framework to build a comprehensive response to trauma is needed for effective implementation.

## Recommendations

▪ To better integrate TIC into everyday practice it should be a prioritization in both operational and professional supervision.
▪ Training in TIC should become mandatory training for all mental health staff to ensure expressed importance of trauma in mental healthcare is reflected in the training provided.
▪ A systematic approach to TIC is required that incorporates staff training, organizations' policy and procedures, environmental considerations, and staff well-being.
▪ A competency framework should be used to inform staff training with all staff being expected to be trauma informed and additional competencies toward being trauma skilled, trauma enhanced or trauma specialist, depending on the staff member's role in the organization. This requires organizations to prioritize and invest in becoming trauma responsive.
▪ Future research on the implementation of TIC into routine practice should involve codesign and participation of all disciplines including people with lived experience and peer workers.

## Data Availability Statement

The raw data supporting the conclusions of this article will be made available by the authors, without undue reservation.

## Ethics Statement

The studies involving human participants were reviewed and approved by Metro South Addiction and Mental Health Human Ethics Committee HREC/2019/QMS/52067. The patients/participants provided their written informed consent to participate in this study.

## Author Contributions

MS, KP, TN, MM, and FD conceived the study. FD, LN, MS, KP, MM, and NS designed the study. FD and LN did the initial thematic analysis and SP reviewed the analysis. FD supervised the whole project. SP was supervisor and consultant on the thematic analysis. All authors were involved in writing the paper.

## Conflict of Interest

The authors declare that the research was conducted in the absence of any commercial or financial relationships that could be construed as a potential conflict of interest.

## Publisher's Note

All claims expressed in this article are solely those of the authors and do not necessarily represent those of their affiliated organizations, or those of the publisher, the editors and the reviewers. Any product that may be evaluated in this article, or claim that may be made by its manufacturer, is not guaranteed or endorsed by the publisher.
